# Lateral confined growth of cells activates Lef1 dependent pathways to regulate cell-state transitions

**DOI:** 10.1038/s41598-022-21596-4

**Published:** 2022-10-15

**Authors:** Luezhen Yuan, Bibhas Roy, Prasuna Ratna, Caroline Uhler, G. V. Shivashankar

**Affiliations:** 1 Division of Biology and Chemistry, Paul Scherrer Institut, 5232 Villigen, Switzerland; 2 Department of Health Sciences and Technology, ETH Zurich, 8092 Zurich, Switzerland; 3 Mechanobiology Institute, National University of Singapore, Singapore, 117411 Singapore; 4 Institute of Molecular Oncology, Italian Foundation for Cancer Research, 20139 Milan, Italy; 5 Massachusetts Institute of Technology, Cambridge, MA USA

**Keywords:** Cell biology, Computational biology and bioinformatics, Stem cells

## Abstract

Long-term sustained mechano-chemical signals in tissue microenvironment regulate cell-state transitions. In recent work, we showed that laterally confined growth of fibroblasts induce dedifferentiation programs. However, the molecular mechanisms underlying such mechanically induced cell-state transitions are poorly understood. In this paper, we identify Lef1 as a critical somatic transcription factor for the mechanical regulation of de-differentiation pathways. Network optimization methods applied to time-lapse RNA-seq data identify Lef1 dependent signaling as potential regulators of such cell-state transitions. We show that Lef1 knockdown results in the down-regulation of fibroblast de-differentiation and that Lef1 directly interacts with the promoter regions of downstream reprogramming factors. We also evaluate the potential upstream activation pathways of Lef1, including the Smad4, Atf2, NFkB and Beta-catenin pathways, thereby identifying that Smad4 and Atf2 may be critical for Lef1 activation. Collectively, we describe an important mechanotransduction pathway, including Lef1, which upon activation, through progressive lateral cell confinement, results in fibroblast de-differentiation**.**

## Introduction

Cells naturally undergo various differentiation, trans-differentiation and dedifferentiation programs within in vivo systems during embryonic development, tissue repair, and cancer progression^1–3^. In landmark experiments, Yamanaka and his colleagues showed that the constitutive expression of Oct4, Sox2, Klf4 and cMyc transcription factors induces the transition of somatic cell states into induced pluripotent (iPSC) states in vitro^4^. However, in vivo, various types of cell-state transitions occur in the absence of exogenous Yamanaka factors, suggesting an important role for the local mechanical microenvironment in the modulation of cell-state transitions. We recently demonstrated that fibroblasts cultured in laterally confined microenvironments may acquire stem-ness like properties after multiple cell divisions^5^. However, the underlying nuclear mechanotransduction pathways driving such cell-state transitions are unknown.

Recent single-cell experiments have revealed how, upon constitutive expression of Yamanaka factors, cells dedifferentiate in three steps: initiation, maturation, and stabilization^6^. In the initiation phase, cells gradually repress somatic cell genes and undergo mesenchymal-to-epithelial transition via complex biochemical regulatory pathways. In the maturation phase, cells rapidly acquire an iPSC gene expression profile, coupled with the sequential activation of Oct4, Nanog, and Sox2^7^. Finally, in the stabilization phase, cells gain an endogenous pluripotency network^8^. Given the important role of reprogramming factors in rewiring the genome and establishing the stem cell gene expression profile, we speculated that in fibroblasts grown under lateral confinement, somatic transcription factors would be required to activate the endogenous factors to induce cell-state transitions. Supporting this hypothesis, previous studies have shown that altering cell geometry can mechanically induce epigenetic modifications in somatic cells and activate downstream transcription factors such as YAP, mRTFA, and p65^9–11^. Since the fibroblast cell-state transitions, using lateral confinement, is controlled by cell geometry changes, we hypothesized that matrix dependent mechanotransduction pathways must exist to ensure the regulation of genome programs.

In this paper, by combining time-lapse RNA-seq analysis with a network optimization method based on Prize-Collecting Steiner Trees, we identify Lef1 dependent pathways as critical regulators for fibroblast cell-state transitions. Using ChIP-qPCR, we show that Lef1 directly interacts with its target promoter during the cell-state alterations. Lastly, we analyze the upstream activation pathways of Lef1, thereby identifying Smad4 and Atf2 as potential upstream regulators. Our studies have important implications in understanding the coupling between nuclear mechanotransduction and cell-state transitions.

## Results

### Global gene expression analysis of fibroblast cells grown under lateral confinement reveals their de-differentiation

**Figure 1:**
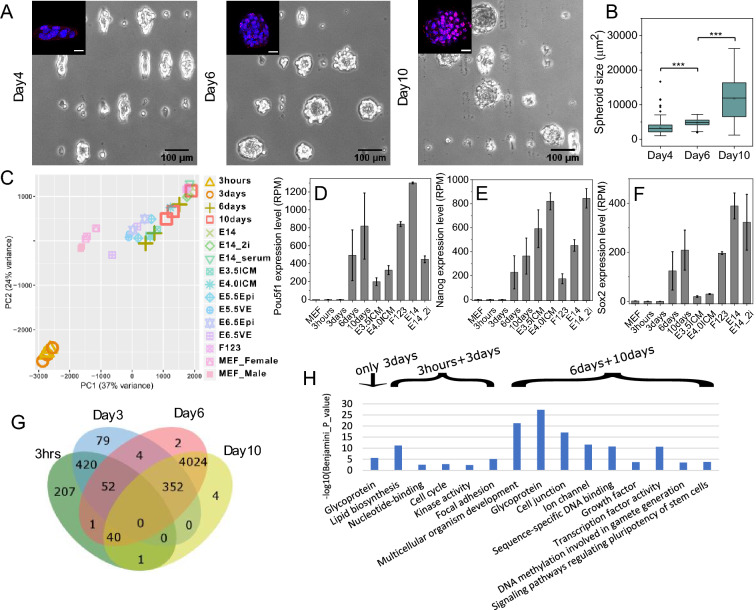
Global gene expression analysis of fibroblast cells grown under lateral confinement reveals their de-differentiation. (A) Differential interference contrast images of cells grown under lateral confinement for 4, 6, or 10 days. The insets show fluorescence images of Oct4 staining (in red) and nuclei (in blue). Inset scale bar is 20 um. (**B**) The box plot of spheroid sizes on days 4, 6, and 10 reveals that cells organize into a spheroid on the micropattern, which progressively increases in size. *** *P* < 0.001; n = 499, 440, 153 for day4, 6, and 10 respectively; two-sided Student’s t-test was used. (**C**) Principal Component Analysis of the gene expression profile shows change of cell states through de-differentiation. Genes involved in this analysis are differentially expressed with adjusted *p* value < 0.01 and |log2 Fold change|> 2. (**D**, **E**, **F**) Bar plot graphs of Oct4, Nanog, or Sox2 gene expression (RPM) over four time points during de-differentiation and how it compares to other cell types. MEF: mouse embryonic fibroblasts; E3.5ICM: E3.5 inner cell mass; E4.5ICM: E4.5 inner cell mass; F123: F123 cell line; E14: ES-E14TG2a cell line cultured in mouse ES cell media; E14_2i: ES-E14TG2a cell line cultured in 2i condition. Error bars represent ± SD. (**G**) Venn diagram showing the number of up-regulated genes over the four time points during de-differentiation. For example, there are 4024 genes up-regulated on day6 and day10 compared to the 3 h and day3 timepoints. Up-regulated genes have FDR (adjusted *p* value) < 0.01. (**H**) Functional annotation of genes that are overexpressed on the day3 timepoint (207 genes), at both 3 h and day3 timepoints (420 genes), and at both day6 and day10 timepoints (4024 genes).

To study the mechanisms underlying mechanically-induced alterations in fibroblasts, we grew mouse embryonic fibroblasts (NIH 3T3 cell line) under lateral confinement in the absence of exogenous biochemical factors as reported earlier^5^: We seeded the cells on 1800 µm^2^ rectangular fibronectin micropatterns (19 µm × 95 µm) on a culture dish in such a manner that each micropattern was occupied by a single cell. Cells were allowed to grow and divide under such lateral confinement for 10 days. After 2 days, cells started to grow into a multi-layer colony. During Day4 to Day6, the colony formed spheroids (Fig. S1). In agreement with previous observations, the size of the spheroids continuously increased during the 10 days of lateral confinement^5^ (Fig. 1A). The spheroid size was quantified by measuring the projected area of an individual spheroid after segmenting it from the background image. After 10 days, these fully grown spheroids (Day10) had an average projected area of about 12,000 µm^2^ and contained approximately 200 cells (Fig. 1B). Consistent with previous observations, Day10 spheroid cells had acquired stem cell-like properties, characterized based on increased Oct4 protein expression^5^ (Fig. 1A: inset). Thus we use Oct4 expression levels as a marker for fibroblast de-differentiation in all of our studies. Oct4 expression colocalized with nuclear staining, although imaging in the Z-plane revealed that this labelling varied for different nuclei (Fig. S3A). To check cell death under the lateral confinement cell culture condition we stained with DRAQ7 (which labels dead cells) and the nuclei with Hoechst. There are almost no dead cells in Day2 colonies and the number of dead cells increased with increasing time from Day4 to Day10 colonies (Fig. S2, Fig. S3B).

Next, we performed time-lapse RNA-seq analysis during the laterally confined growth of fibroblasts at four timepoints: 3 h, 3 days, 6 days, and 10 days. To identify differentially expressed genes between these four timepoints, the RPM (Reads Per Million) values for all genes were used as the input to the DESeq2 software and genes with a false discovery rate (FDR) lower than 0.01 were identified as differentially expressed (DE genes). The characteristic mesenchymal fibroblast transcriptome was maintained at 3 h when cells were elongated on the micropattern, but the transcriptome started to change on day 3 as the cells began to form spheroids on the fibronectin micropattern (Fig. 1C). The most significant change of the transcriptome occurred from day 3 to day 6, when we identified approximately 4000 genes that were up-regulated with a fold change of more than 4 (FDR < 0.01). From day 6 to day 10, less than 100 DE genes were detected although cells had been changed into mouse ES medium, suggesting that cell-state transition process was already initiated by day 6. While the expression levels of the stem cell markers Oct4, Nanog, and Sox2 increased significantly from day 3 to day 6 and increased further on day 10, their expression on day 6 and day 10 was closer to previously published mESC data than the fibroblast cells (see Methods) (Fig. 1D–F). To reveal changes in cellular pathways and functions during such de-differentiation process, the DE gene lists from the four timepoints (from Fig. 1G) were annotated and clustered using the DAVID tool^12^ (Fig. 1H). Within the 420 DE genes down-regulated in both the day 6 and day 10 timepoints, 26 of them were focal adhesion genes, including talin (Tln1) and integrin (e.g. Itga2, Itgav), thereby indicating that the cells were gradually losing fibroblast properties while forming spheroid-like colonies. Of the 4000 DE genes up-regulated in later timepoints (day6 and day10), over 200 transcription factors were annotated, including POU domain proteins (Pou2f1, Pou3f1, Pou5f1, etc.), Kruppel-like factors (Klf2, Klf5, Klf11, etc.), and SOX gene family proteins (Sox2, Sox3, Sox6, etc.), thereby suggesting that cells gained stem cell-like properties, which was consistent with the Oct4 staining (Fig. 1A: inset). In order to infer cell type changes that may take place over the course of the 10-day culture period, we included published RNA-seq datasets from other cell types in mice for comparison (16 conditions, 38 samples; see supplementary information)^13–16^. Focusing on the 3959 DE genes (FDR < 0.01 and |log2 Fold change|> 2) identified in our RNA-seq dataset and reducing the dimensions further from 3959 to 2 through a principal component analysis (PCA), we visualized the distance between the different samples (Fig. 1C). This revealed that global gene expression at the day 6 and day 10 timepoints were closer in expression to mouse embryonic stem cells. Collectively, these results suggest that cell growth under lateral confinement could de-differentiate fibroblasts (NIH 3T3 cell line), and that the most dramatic alterations in their gene expression occurs between day 3 and day 6 of confinement.

### Prize-collecting Steiner tree analysis highlights Lef1 dependent signaling during lateral confined growth of fibroblasts

**Figure 2:**
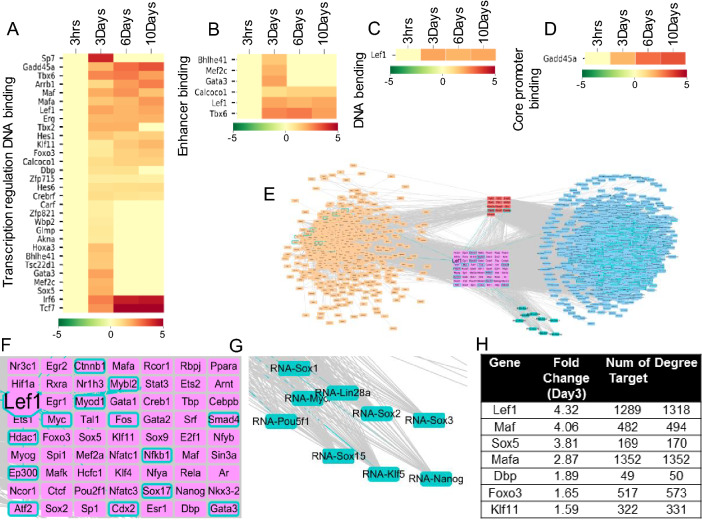
Prize-Collecting Steiner Tree analysis highlights Lef1 dependent signaling during lateral confined growth of fibroblasts. (**A**–**D**) The heatmaps show the expression changes of transcriptional regulators which were found by gene ontology annotation. They contain the terms GO:0,044,212 (transcription regulatory region DNA binding), GO:0,035,326 (enhancer binding), GO:0,008,301 (DNA binding and bending), and GO:0,001,047 (core promoter binding). The values refer to the log2 fold change compared to the 3 h sample. (**E**) Transcriptional regulatory network derived using the Prize-Collecting Steiner Tree method. Nodes in orange, red, and pink represent genes up-regulated on day3 and important intermediates that connect them. Nodes shown in red and pink are transcriptional regulators. Nodes in blue and turquoise represent genes up-regulated on day6. Within these, turquoise nodes represent reprogramming factors. (**F**, **G**) Enlarged pictures of (**E**) showing transcriptional regulators (in pink and Lef1 and its interactors with turquoise border) that can regulate the expression or bind to the gene loci of reprogramming factors (in turquoise). (**H**) Ranking of the transcriptional regulators (pink nodes in (**E**)).

To identify regulatory mechanisms that lead to gene expression changes from day3 to day6, we analyzed the functions of the genes up-regulated on day3, compared to 3 h. Our hypothesis was that genes up-regulated on day3 could be important for the gene expression changes occurring between day3 and day6. We annotated the biological processes and molecular functions of the 675 up-regulated DE genes on day 3 using the gene ontology enrichment tool, pantherdb^17^. Up-regulated genes were linked to processes involved in the regulation of cell motility, proliferation, and histone acetylation (FDR < 0.05). Among the 675 up-regulated DE genes there were thirty transcription factors (including Lef1 and Klf11), and 6 of which had enhancer binding capability (Fig. 2A–D). Next, we quantified the expression changes in known reprogramming factors^8^ (including Nanog, Sox2, and Oct4) at later time points and found that all but c-Myc showed significant up-regulation at either the day6 or day10 timepoint (Fig. S4A). While it is likely that the switch to de-differentiated gene expression profile on day6 may be due to the expression of reprogramming factors, the mechanisms underlying the mechanical activation of such reprogramming factors is unclear.

Next, we constructed a comprehensive transcriptional regulatory network to focus on potential regulatory events between day3 and day6. In order to include all possible transcriptional regulation pathways, we collected protein–protein interaction (PPI) data from the STRING database (containing 15,564 proteins and 975,722 interactions) and transcription factor (TF)-target gene relationships from 13 sources containing 387 TFs and 19,790 protein-coding gene targets (see Methods). The Prize-Collecting Steiner Tree network analysis method was used to derive a subnetwork that could explain the gene expression changes of the de-differentiation process^18^ (Fig. 2E). Firstly, this method evaluates the changes in gene expression detected between the 3 h and day3 timepoints., represents the identified proteins as nodes (orange, red and pink colored nodes in the left and middle of the network), and links them based on the PPI data. Secondly, genes up-regulated from the day3 to day6 timepoints were added to the network as RNA nodes (blue and turquoise colored nodes in the right side of the network) and linked based on TF-target gene relationships. A key benefit of this method is that important intermediate genes are also included in the network, even if no expression changes are detected, e.g. Beta-catenin and Smad4 (see Methods). Specifically, this method found optimized trees from the global interactome (combined PPI and TF-targets) with gene expression changes as node weight. Then we constructed the transcriptional regulatory network by connecting edges in the optimized trees based on the global interactome.

In the constructed network, transcriptional regulators were defined as protein nodes (red and pink colored) that had direct relationships with RNA nodes. These transcriptional regulators included transcription factors, such as Lef1 and Myc, and genes involved in epigenetic modification, such as Ep300 and Hdac1. Among the transcriptional regulators we identified those (pink colored nodes, Fig. 2F) that had reprogramming factors as targets (turquoise colored nodes, Fig. 2G). Ranking this subset of transcriptional regulators based on their gene expression changes between the 3 h and day3 timepoints we identified Lef1 as a potential key regulator of fibroblast de-differentiation: it was not only highly up-regulated, but also found to potentially control thousands of genes that get up-regulated from day3 to day6 (Fig. 2H). Lef1 up-regulation before the up-regulation of reprogramming factors suggests the presence of an early dedifferentiation stage when cells prepare for the expression of reprogramming factors. To further elucidate the role of Lef1, we analyzed its relationship with other transcriptional regulators: 15 out of 76 transcriptional regulators, including Beta-catenin, Smad4, and Ep300 physically interacted with Lef1. In addition, 38 transcription regulators were potential Lef1 targets (red and pink colored nodes with blue borders in Fig. S4B). Collectively, these findings suggest that Lef1 is a key TF in the early de-differentiation induced by the lateral confinement and growth of fibroblasts.

### Knockdown of Lef1 and time course studies of Lef1 and Oct4 show the critical role of Lef1 in fibroblast de-differentiation

**Figure 3:**
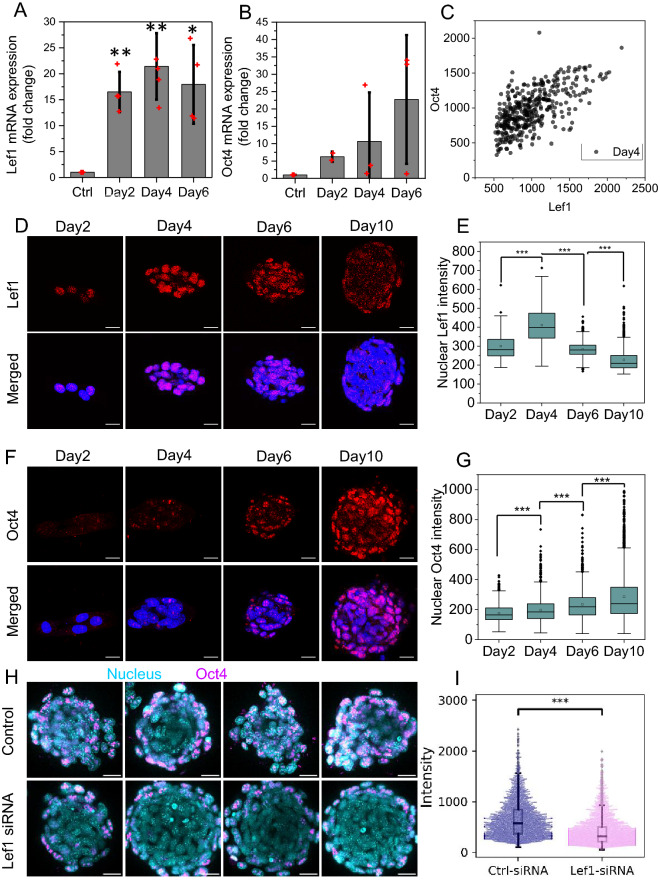
Knockdown of Lef1 and time course studies of Lef1 and Oct4 show the critical role of Lef1 in fibroblast de-differentiation. (**A**, **B**) Bar plot graphs of Lef1 and Oct4 mRNA expression level over time, error bars represent ± SD. (**C**) The scatter plot of Lef1 and Oct4 nuclear fluorescence intensity. Each dot represents the average intensity for one nucleus. The Pearson correlation coefficient r is 0.64. (D) The fluorescence images of Lef1 (in red) and nucleus (in blue) for cells at day2, 4, 6, and 10. Scale bar is 20 um. (**E**) The box plot shows the quantification of average nuclear Lef1 intensity over time; n = 244, 279, and 673 respectively. (**F**) The fluorescence images of Oct4 (in red) and nucleus (in blue) for cells at day2, 4, 6, and 10. Scale bar is 20 um. (**G**) The box plot shows the quantification of average nuclear Oct4 intensity over time; n = 481, 1486, 1248 and 2472 respectively. (**H**) The fluorescence images of Oct4 (in magenta) and nucleus (in cyan) for control and Lef1 siRNA treated cells. Scale bar is 50 um. (**I**) Quantification of Oct4 nuclear intensity shows distinct distributions for control and Lef1 siRNA samples; *** *P* < 0.001; n = 2708 and 3171 respectively.

To dissect properties of the early de-differentiation, we focused on the dynamic changes in the transcription factor Lef1 and the stem cell marker Oct4. We measured the RNA levels of these two genes with quantitative PCR. In line with the RNA-seq data, Lef1 had significantly higher expression levels on days 2, 4, and 6 than control cells in 2D culture (Fig. 3A). Interestingly, Oct4 was also upregulated starting from day4 (Fig. 3B). To visualize the heterogeneity of marker expression within the cell population on day4 (early de-differentiation stage), we immunostained for Lef1 and Oct4 (Fig. 3D, F). To measure the fluorescent intensity, each DAPI-stained nucleus was segmented in 3D from the background and separated from other nuclei. This was then used as a mask to measure the Lef1 and Oct4 concentration for each nucleus (see Methods). We found an increase in the abundance of nuclei with a high Oct4 concentration (defined by an average Oct4 fluorescence intensity of over 300 per pixel), during the de-differentiation process. Specifically, 10% of all nuclei showed high concentration of Oct4 on day4, 20% on day6, and 22% on day10 (Fig. S5). On day6 we also started to observe a significant increase in the population mean of the Oct4 signal (Fig. 3G). In terms of Lef1 staining, we found that a significant increase occurred on day4 as compared to the earlier time points (Fig. 3E). We also observed a correlation between nuclear Oct4 and Lef1 intensity when we co-stained these two proteins (Fig. 3C).

To validate the role of Lef1 in the de-differentiation process, we knocked down the expression of Lef1 using siRNA. Addition of Lef1 siRNA 2 h after seeding the cells resulted in a significant reduction in Lef1 nuclear fluorescence intensity for up to 5 days after seeding (Fig. S6A, B, C) compared to cells without Lef1 siRNA. We examined the de-differentiation efficiency through immunostaining of Oct4 at day10. The addition of negative control siRNA (NC siRNA) did not affect the efficiency (Fig. S6D). Also the distribution of Oct4 nuclear intensity for cells with or without NC siRNA treatment was almost identical and no significant difference was detected for the mean of Oct4 intensity between these two conditions (Fig. S6D). However, the Lef1 siRNA knockdown cells exhibited significantly lower Oct4 intensity compared to those treated with NC siRNA (Fig. 3H,I and Fig. S6E,F). The distribution of Oct4 nuclear intensity between these two conditions was highly distinct, with a larger portion of nuclei expressing high Oct4 staining following NC siRNA treatment as compared to Lef1 siRNA treatment. In addition, using qPCR, we found that Oct4 mRNA levels reduced under Lef1 siRNA treatment (Fig. S7D). Besides, we also performed alkaline phosphatase staining (as described earlier in reference 5) to check the stem cell-like properties. We found a significant reduction in spheroid number under Lef1 siRNA treatment (Fig. S7A–C). The ratio of positively stained spheroid dropped significantly under Lef1 siRNA treatment. These results suggest that Lef1 is an important regulator in the fibroblast de-differentiation process.

### Lef1 binds to the gene loci of the reprogramming factors during the mechanically induced fibroblast de-differentiation

**Figure 4:**
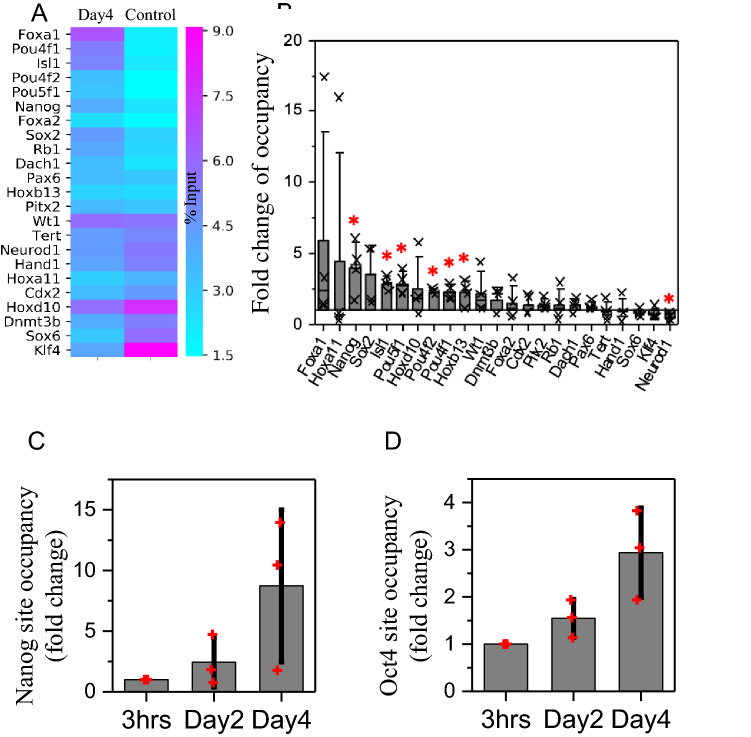
Lef1 binds to the gene loci of the reprogramming factors during the mechanically induced fibroblast de-differentiation. (**A**) The heatmap shows binding of Lef1 to the promoter regions of selected genes in Day4. Control cells grown on 2D culture are also shown. The unit is percentage of input. (**B**) Fold change of Lef1 promoter occupancy comparing Day4 sample to control sample. *P < 0.05. (C/D) The bar plot shows the increase of binding of Lef1 to Nanog and Oct4 promoter regions at 3 h, Day2 and Day4.

Next, we studied whether Lef1 could directly regulate the expression of reprogramming factors. We used the commercially available Chromatin Immunoprecipitation (ChIP) qPCR primer array designed for the promoter regions of mouse stem cell transcription factors (see Methods). This array contains homeobox genes, GATA binding proteins and TFs that could induce de-differentiation. Based on our collected TF-target gene relationship data, 23 of the included genes were potential targets of Lef1. Using the ChIP-grade antibody (Merck 17–604) to pull down Lef1 along with the genomic DNA fragments bound to it, we studied which of these stem cell transcription factors were directly targeted by Lef1. This analysis identified 6 genes; In particular, we found that Nanog and Oct4 (Pou5f1) showed reproducibly higher promoter occupancy in the early de-differentiation stage (Fig. 4A,B). We confirmed this result by designing primers to the Nanog and Oct4 promoter regions and investigating promoter occupancy by ChIP-qPCR (Fig. 4C,D). We found that Lef1 increased its binding to the promoter regions of Nanog and Oct4, during the lateral confinement culture from Day2 to Day4. We also checked the correlation between Lef1 spatial distribution and histone modifications. Lef1 significantly decreases its colocalization with the inactive mark H3K9me3 and significantly increases its colocalization with the active mark H3K4me3 on Day4 (Fig. S8A, B). We also found that Lef1 significantly increases its colocalization with RNA pol II (Fig. S8C). Collectively, these results suggest that Lef1 directly regulates the expression of the key reprogramming factors Oct4 and Nanog in fibroblast de-differentiation.

### Lef1 activation is potentially regulated by Smad4 and Atf2 pathways

**Figure 5:**
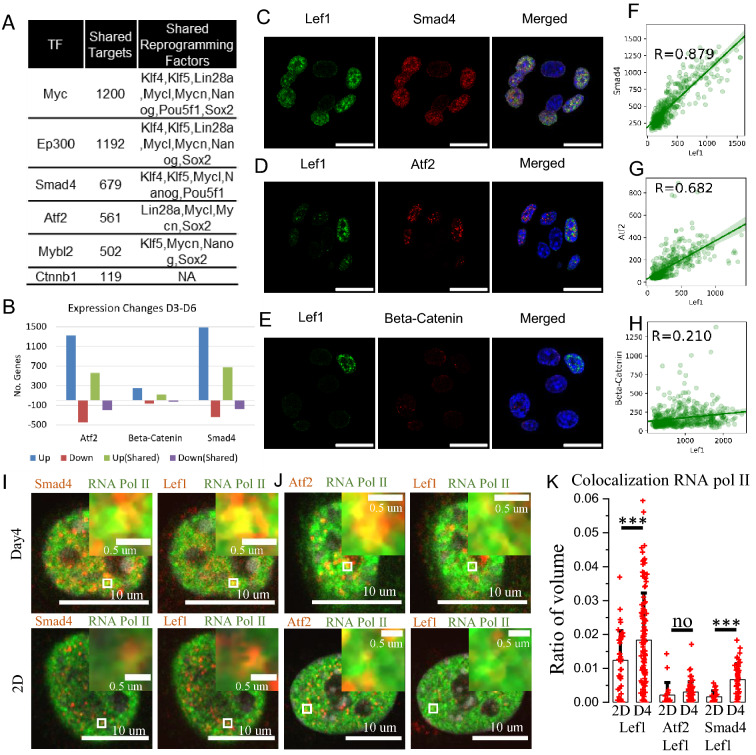
Lef1 activation is potentially regulated by Smad4 and Atf2 pathways. (**A**) Table of identified genes that could interact with Lef1, the number of shared targets with Lef1 and the shared reprogramming factors as targets. (**B**) Bar plot showing the number of genes up-regulated or down-regulated from day3 to day6. Up (Shared): shared targets with Lef1 that are up-regulated. Down (Shared): shared targets with Lef1 that are down-regulated. These targets are differentially expressed with adjusted *p* value < 0.01. (**C**, **D**, **E**) The staining of Lef1, Smad4, and Nucleus; Lef1, Atf2 and Nucleus; and Lef1, Beta-catenin and Nucleus in day4 sample. Scale bar is 20 um. (**F**, **G**, **H**) Scatter plots showing the averaged nuclear fluorescence intensity of Lef1 and Smad4; Lef1 and Atf2; and Lef1 and Beta-catenin along with linear fits and the corresponding Pearson correlation coefficients. Representative images of co-staining with Lef1/Smad4/RNA pol II (**I**), Lef1/Atf2/RNA pol II (**J**). The insets are the zoom of small white box regions from the respective images. (**K**) Barplot shows the change of colocalization from 2D culture conditions to D4 (Day4 sample). The unit is the ratio of colocalized volume and the nucleus volume. Each dot represents one nucleus. The first two bars are the colocalization of Lef1 and RNA pol II. The middle two bars are the colocalization of Lef1, Atf2 and RNA pol II. The last two bars are the colocalization of Lef1, Smad4 and RNA pol II.

To dissect the molecular mechanisms regulating the activation of Lef1 during the early de-differentiation stages, we studied the expression and localization of potential Lef1 activators. Previous studies suggested that Lef1 requires association with other DNA binding proteins for transcriptional regulation^19,20^. Hence, we focused our investigation on transcriptional regulators that can interact with Lef1. Based on the collected TF-target gene relationships and constructed transcriptional regulation network mentioned above, we identified 15 transcriptional regulators that not only interacted with Lef1 but also shared many up-regulated targets at day6 as compared to day3 (Fig. 5A,B, the full list is shown in Fig. S9A). Three of the identified regulators, Smad4, Atf2, and Beta-catenin, can activate Lef1 transcription^21–23^. Interestingly, only Smad4 and Atf2 had Nanog, Oct4 or Sox2, as shared targets with Lef1 (Fig. 5A). We immunostained Lef1 with the three identified regulators in cells from the day4 timepoint to observe their localization. Smad4 and Atf2 both co-localized with Lef1 in the cell nucleus, whereas Beta-catenin remained mostly in the cytoplasm (Fig. 5C,D,E). This co-localization was quantified at the cellular level by segmenting individual nuclei using the DAPI staining, followed by calculation of the average fluorescence intensity for each protein of interest. The correlation was 0.88 between Smad4 and Lef1 and 0.68 between Atf2 and Lef1, while the correlation between Beta-catenin and Lef1 was only 0.2 (Fig. 5F,G,H). These results suggested that Lef1 was more likely to be associated with Smad4 and Atf2 than with Beta-catenin in the de-differentiation stage. However, quantifying the nuclear fractions of Beta-catenin will not be sufficient to exclude the roles of Beta-catenin in Lef1 dependent signaling. To explore the role of Lef1 association with Atf2 or Smad4, we costained Lef1/Atf2/RNA pol II or Lef1/Smad4/RNA pol II. We found that RNA pol II tends to significantly localize to the Lef1 and Smad4 on Day 4 (Fig. 5I–K). Further, using qPCR, we found that combined knock down of Lef1, Atf2 and Smad4 showed increased reduction of Oct4 mRNA levels compared with Lef1 knockdown alone (Fig. S7D). To further test the role of Beta-catenin in Lef1 activation, we blocked the interaction between Lef1 and Beta-catenin using the inhibitor iCRT3. Interestingly, iCRT3 inhibition led to a significant increase in de-differentiation, as measured with Oct4 staining at the Day10 timepoint, suggesting that the Beta-catenin pathway may play a distinct role in the mechanically induced fibroblast de-differentiation (Fig. S9B, C). Collectively, these results identified Smad4 and Atf2 as potential activators of Lef1.

## Discussion

**Figure 6:**
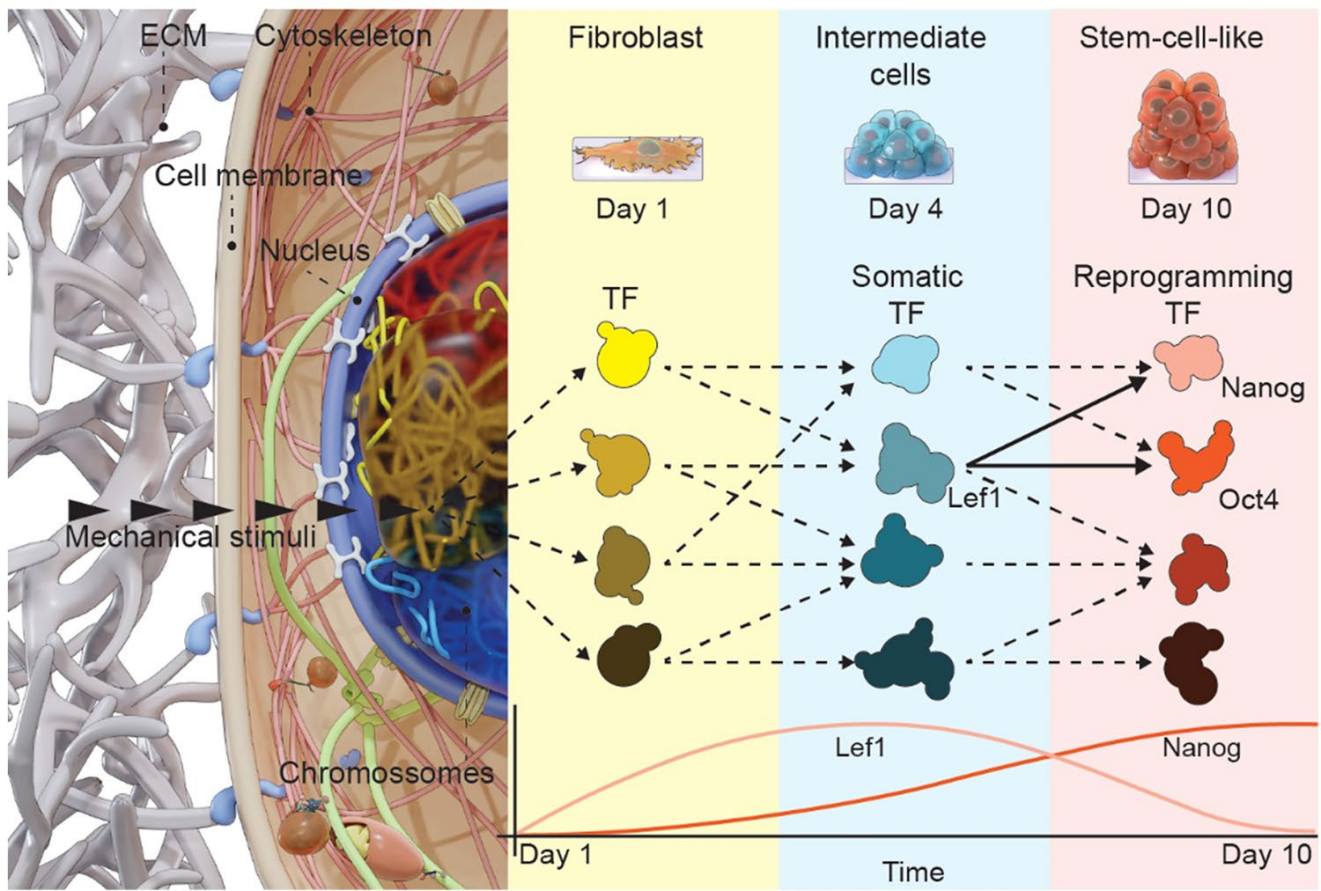
Schematic representation of the molecular mechanism of laterally confined growth induced fibroblast de-differentiation.

The ability to induce nuclear reprogramming either by expressing exogenous transcription factors or using small molecules has revealed the plasticity of cell-states^8^. In addition, recently it has been shown that cell-state transitions can also be induced by mechanical modulations of the extracellular environment alone, without any exogenous transcription factors^5^. The geometric constraints on the cells imposed by the micropatterns (that provide the boundary conditions) during the lateral confined growth result in the alteration in the cell mechanical and transcriptional state. This suggests the presence of a tight coupling between the extracellular mechanical environment and somatic transcription factors that can activate mechanotransduction pathways to induce cell-state transitions (Fig. 6).

By obtaining time-course RNA-seq data, we identified candidate key somatic factors responsible for the mechanical induction of fibroblast cell-state transitions. This was achieved by combining the RNA-seq data together with the protein interaction database STRING and the transcription factor-target gene databases TRANSFAC and JASPAR, and collectively analysing this data using the Prize-Collecting Steiner Tree network optimization method^6,24,25^. The gene ontologies data (Fig. 2A–D) provides candidates of transcriptional regulators shown upregulation on Day3. Since some of the potentially important transcriptional factors enhancing dedifferentiation may not show up in RNAseq data, we used the Prize-collecting Steiner tree method to identify them. This method combined PPI and TF-target datasets to identify transcription factors when three time points of RNAseq data are considered. The genes upregulated on Day3 which appears as protein nodes can link genes upregulated on Day6 which appears as RNA nodes through transcriptional regulators which may or may not show expression changes. Lef1 was selected based on the expression fold change and number of upregulated downstream targets as shown in Fig. 2H. In addition, we were also able to identify Lef1’s potential coactivators Atf2/Beta-Catenin/Smad4 which do not show expression changes. We validated the role of Lef1 by showing that downregulation of Lef1 decreases the efficiency of fibroblast cell-state transitions. In addition, many reprogramming genes are potential targets of Lef1. Recent studies have also shown that Lef1 can enhance oncogenic effect in hepatocellular carcinoma (HCC) cells through Notch signalling pathway^26^. In addition, overexpression of Lef1 promotes the tumorigenicity of Esophageal squamous cell carcinoma (ESCC) through the TGF-β signaling pathway^27^. Furthermore, Lef1 has been shown to regulate Nanog and Oct4 expression in mouse embryonic stem cells^28,29^. Earlier experiments related to lateral inhibition in cell specification mediated through mechanical signalling highlighted the role of YAP and TAZ^30^. However, in our experiments, we did not observe the changes in YAP and TAZ gene expression in 3 Days cultures compared to the control, and they were also not observed in the network model (Fig. 2E). Notch4 was upregulated on Day 3 and showed interactions with Atf2 and Beta-catenin, suggesting that Notch4 could be an upstream factor of Lef1 activation. Since the dedifferentiated cells tend to locate at the outer layer of the colonies, the cell state transitions could be driven by both mechanical as well as spatial signals. The outer cells, during lateral confined growth, are in privileged positions without being fully surrounded by other cells which could also be a potential spatial signal for outer cells to undergo dedifferentiation. Collectively, these studies suggest that Lef1 could enhance dedifferentiation through distinct pathways in different functional contexts including the lateral confined dedifferentiation of fibroblast.

Furthermore, using the ChIP-qPCR promoter occupancy assay, we found that Lef1 not only localizes in the nucleus, but also directly binds to promoter sites of Nanog and Oct4, two prominent reprogramming factors. Consistent with this, time course analysis of Lef1, Oct4 and Nanog suggests that the activation of Lef1 induces the downstream expression of Nanog and Oct4. These results indicate that the somatic transcription factors are activated by the local mechanical environment, followed by a biphasic transition in gene expression and subsequent induction of the reprogramming factors. This highlights the important role of somatic transcription factors for mechanically induced cell-state transitions.

To investigate the molecular pathways leading to the activation of Lef1, we analyzed the role of the NFkB, Beta-catenin, Smad4 and Atf2 pathways. Earlier studies have shown that NFkB is sensitive to local mechanical perturbations and can act as an upstream transcription factor for Lef1^11,31^. We found that the inhibition of p65 (an NFkB subunit) using SN50 reduced the de-differentiation efficiency (Fig. S9D). Such an effect could result from NFkB inhibiting Lef1 or directly controlling gene expression of downstream factors. In addition, Beta-catenin could bind to and activate Lef1 through the Wnt signalling pathway, and such activation could be facilitated by cadherin junctions^32–34^. To investigate these hypotheses, we analyzed the time course correlation between Beta-catenin and Lef1, finding only weak correlation with their nuclear localization. Moreover, inhibiting their physical interaction indicated no significant coupling between Beta-catenin and Lef1 in regulating the downstream pathways. However, the Wnt signaling pathway is up-regulated during this reprogramming process, suggesting that Lef1 role maybe independent of Beta-catenin/Wnt signaling pathway (Fig. S10F). Previous studies suggested that Lef1 can switch partner from Beta-catenin to other activators^35^. Interestingly, careful analysis of the upstream interaction partners of Lef1 identified Smad4 and Atf2 as potential candidates for the regulation of Lef1 activation during the fibroblast cell-state transitions. In fact, previous studies have shown that Smad4 and Atf2 can activate Lef1^22,23^. Consistent with this, we found that Smad4 and Atf2 were colocalized with Lef1 in the nucleus during mechanically induced cell-state transitions. The TGF-beta/SMAD pathway has been shown to be regulated by mechanical signals in the environment, such as ECM perturbations^36^. In addition, activated Smad proteins can interact with different DNA binding cofactors, which might include Lef1, to target a unique group of genes^37^. These results collectively suggest that Lef1 dependent pathways, in consort with Atf2/Beta-Catenin/Smad4, are transiently activated to regulate the downstream transcription factors. The non-monotonic kinetics of Lef1 might be determined by the differential role of upstream signaling, e.g. Wnt pathway, in dedifferentiation process as reported in some studies^38^. Since the fibroblast identity decreased and the cell–cell contacts formed, we also explored the potential role of cytoskeleton related processes (Fig. S10D,E). We found a high correlation between nuclear Lef1 level and cytoplasmic GEF-H1 level (Fig. S10A–C). Taken together, these results indicate that multiple mechanotransduction pathways could activate Lef1 to induce the expression of downstream transcription factors.

Collectively, our paper shows that in the absence of exogenous factors, the local mechanical microenvironment can activate cell-state transitions through Lef1 dependent signalling pathways. Our Prize-Collecting Steiner Tree analysis also reveals other somatic transcriptional regulators that could be critical for the induction of cell-state transitions. Our results suggest that somatic cells in vivo downregulate such key somatic transcription factors or their regulatory pathways in order to maintain cellular homeostasis, since the aberrant activation of these transcriptional regulators over multiple cell divisions in vivo may serve as precursors for the induction of dedifferentiation and transdifferentiation pathways. Since we show that the induction of fibroblast dedifferentiation requires a biphasic cell-state transition, we predict that such cell-state transitions, while rare, could not only induce dedifferentiation programs but also lead to various disease states.

## Methods

### Microcontact printing

PDMS stamps were made as described previously^11^. We stamped fibronectin (0.1 mg/ml working solution, Sigma F1141) on an uncoated hydrophobic polymer dish (ibidi 81,151) to create 19*95 um (~ 1800 um^2) rectangular fibronectin islands. To prevent cells attaching to regions outside the fibronectin islands, the dish was passivated with 0.2% pluronic acid for 5 min and washed with PBS three times.

### Lateral confinement growth of NIH 3T3 cells

Fibroblast growth was performed as per the protocol described previously^5^. NIH3T3 cell is obtained from ATCC. Standard NIH3T3 cell culture medium we used was DMEM high glucose (Gibco, Thermo Fisher Scientific 11,965,092) supplemented with 10% (vol/vol) FBS (Gibco; Thermo Fisher Scientific 16,000,044) and 1% by volume penicillin–streptomycin (Gibco; Thermo Fisher Scientific 15,140,122). Mouse ES medium was from Merck Millipore (ES-101-B). Cells between passages 10 and 20 were used in this study. Cells were maintained at 37 °C in a humidified atmosphere with 5% CO2. Individual NIH3T3 fibroblast cells (at 7000 cells per mL concentration) were seeded on the stamped 1800 um^2^ fibronectin islands. Unattached remaining floating cells were washed off around 30 min later. Standard NIH3T3 cell culture medium was used for 6 days, which was then replaced by mouse ES medium. Both types of medium were changed during cell culture every 48 h. To block the interaction between Beta-catenin and TCF family transcription factors, 25 uM iCRT3 was used (using the same volume of DMSO as control).

### Quantitative real-time PCR (qRT-PCR)

We used qRT-PCR to quantify mRNA levels of genes of interest. After removing the cell culture medium, cells were lysed and total RNA was isolated with RNeasy Plus Mini Kit (Qiagen 74,134). Reverse transcription was carried out on a thermal cycler (Bio-rad C1000) with an iScript cDNA synthesis kit (Bio-rad, 1,708,891). Subsequently, quantitative PCR was performed on the real-time PCR system (Bio-rad CFX96) for 40 cycles with Evagreen supermix (Bio-rad 1,725,200). The expression fold changes of mRNA levels (compared to the control sample) was calculated by ΔΔCt methods with respect to Polr2a levels. The primer sequences used are listed in SI Appendix, Table 1.

### RNA interference

Lef1 siRNA and control siRNA (Dicer-Substrate siRNA) were designed by Integrated DNA Technologies. Sequences of siRNAs used in this study are listed in SI Appendix, Table 4. Transfection was done using lipofectamine 3000 (Thermo fisher L3000015) along with reduced serum medium (Opti-MEM, Thermo Fisher 31,985,070). Cells seeded for 3 h with 1 mL cell culture medium in ibidi dishes were treated with 1.5 uL lipofectamine and 10 pmol siRNA. We followed the same medium changing rate as described under "Lateral confinement growth of NIH3T3 cells".

### Immunostaining

Cells were cultured in polymer dishes as described previously^5^**.** Cells were fixed for 20 min using 4% (by weight) formaldehyde solution (Sigma-Aldrich 252,549). Subsequently, cells were permeabilized with 0.5% by volume Tween 20 (Sigma-Aldrich P1379) in PBS for 20 min, and then blocked with 1% by weight bovine serum albumin (BSA, Sigma-Aldrich A7906), 22.5 mg/mL glycine in PBST (0.1% by volume Tween 20 in PBS) for 30 min. Cells were incubated with the primary antibodies diluted in 1% BSA in PBST at 4 °C overnight. After that, cells were incubated with the secondary antibodies in 1% BSA at room temperature for 1 h. Finally, the nuclei were stained with DAPI or Hoechst (Thermofisher Scientific R37606 or R37605) fluorescent dyes. The primary antibody used: Atf2 (abcam ab32019), Beta-Catenin (abcam ab19381), GEF-H1 (abcam ab155785), Lef1 (abcam ab137872), Oct4 (abcam ab19857), RNA pol II (abcam ab5408), Smad4 (CST 46,535).

### Imaging and image analysis

Samples were imaged using Nikon A1R or Nikon A1 confocal laser scanning microscopes. Confocal images were acquired with a 20X 0.75 NA objective, a 60X 1.4 NA oil objective, or a 100X 1.4 NA oil objective. Confocal z-stack images were captured with 1 or 2 micron z steps; z-depth was no more than 60 microns from the bottom of the dish. DIC images were also acquired using the Nikon A1 confocal microscope. To measure nuclear fluorescence intensity for proteins of interest, each DAPI-stained nucleus was segmented in 3D from the background and separated from other nuclei. Rough 3D segmentations were obtained by combining local and global Otsu thresholding in 2D for all z-planes. For each segment in each z-plane, watershed segmentation was used to separate spatially overlapping nuclei (due to imaging resolution limitation), using a rough nuclear diameter of 10 microns. An in-house software was used to link the segmented 2D nucleus planes in the z-axis through finding the segments with closest center distance. From the distribution of closest center distance, a threshold of 1 micron was selected to determine whether two 2D segments belonged to the same or different nuclei in neighboring z-planes. A second threshold of 100 cubic microns was used to filter out 3D segments derived from the previous steps with too small volumes. Fluorescence intensity within the nucleus was normalized to the volume of the segmented 3D nucleus. We also used the Imaris software to obtain nuclear fluorescence intensities for proteins of interest. To quantify spheroid size, individual spheroids were segmented from the background using green auto-fluorescence images and the projected areas were measured. For spheroid size measurements, images were acquired on a Nikon A1 confocal microscope with pinhole size 8 Airy units (AU).

### ChIP-qPCR and Epitect assay

ChIP-qPCR was performed as described previously^5^. Cells were crosslinked with formaldehyde and the reaction was quenched with glycine. Cells were lysed and chromatin was digested with HindIII restriction enzyme (Thermo Fisher FD0505). ChIP grade anti-Lef1 mouse antibody (Merck 17–604) was coupled with Dynabeads coated with sheep anti-mouse antibody (ThermoFisher 11201D). Beads were added to chromatin and bound to DNA associated Lef1. After this immunoprecipitation (IP) step, precipitated chromatin was treated with Proteinase K (Thermo Fisher 4,333,793). DNA was isolated using sodium acetate method. PCR primers were obtained either from the Epitect array (Qiagen 334,211) or as reported in the SI Appendix Table 1.

### RNA-seq analysis

The RNA-seq data was generated in a previous study carried out in our lab^5^. The study was carried out under four conditions: 3 h, 3 days, 6 days and 10 days after seeding cells. Each condition had three biological replicates and each biological replicate had four technical replicates. We used the sequence alignment software tophat (v2.1.1) to map sequencing reads to the mouse genome, which was downloaded from Ensembl (GenBank Assembly ID GCA_000001635.8) with default parameters^39,40^. Genome annotation files used in this step were also downloaded from Ensembl. The four technical replicates were combined after sequence alignment. The cufflinks (v2.2.1) software was used to calculate the number of reads for each RNA isoform^41^. To calculate the count number for each gene, we combined the number of reads for RNA isoforms from the same gene and derived reads per million (RPM) values. These values were used in the differential gene expression analysis tool DESeq2^42^ (v1.20.0). Genes showing significant differential expressions had adjusted p values (Benjamini–Hochberg) below 0.01. The additional RNA-seq datasets used for the analysis were downloaded from the NCBI-SRA database; details of their accession IDs are listed in SI Appendix Table 3.

### Prize-collecting Steiner tree analysis

Prize-Collecting Steiner Tree is a network analysis method for identifying candidates of critical functional contexts for particular genes of interest within the global interaction network^18^. The input to this method is an interaction network together with values assigned to the nodes (i.e., prizes) and edges (i.e., costs). To represent the transcriptional regulatory pathways in mouse (*Mus Musculus*), we used protein–protein interaction (PPI) data from the STRING database (containing 15,564 proteins and 975,722 interactions) as well as transcription factor (TF)-target gene relationships from 13 sources (listed in SI Appendix, Table 2) including the TRANSFAC and JASPAR databases (containing 387 TFs and 19,790 protein-coding gene targets collected from the Harmonizome and Enrichr database)^25,43^. Proteins from the PPI datasets and TFs from the TF-target gene relationships are represented as protein nodes. Target genes in TF-target gene relationships are represented as RNA nodes. Prizes for protein nodes are defined as log2 fold change in gene expression level comparing 3 days to 3 h. Prizes for RNA nodes are defined as log2 fold change in gene expression level comparing day3 to day6. Costs of edges represent the reliability of the relationships as defined in the PPI dataset. The Prize-Collecting Steiner Tree method determines a tree that maximizes the prizes of the nodes in the tree minus the costs of the edges in the tree. Combined, this allows prediction of the underlying transcriptional regulatory network.

### Statistics and reproducibility

All boxplots are expressed as mean ± SD. Box shows the 25–75 percentage in range and whiskers represent 1.5 × interquartile range. Each experiment was performed in at least three repeats. We evaluated the significance level of the difference in mean using Student’s unpaired two-tailed t-test, performed between the sample of interest and the corresponding control. *P < 0.05; **P < 0.01; ***P < 0.001.

## Supplementary Information

Supplementary Information.

## Data Availability

No sequencing datasets were generated during the current study. Sequencing data analyzed during this study were accessible in NCBI SRA database through the following accession numbers: SRR3083897, SRR3083898, SRR3083901, SRR3083902, SRR3083903, SRR3083904, SRR3083905, SRR3083906, SRR3083907, SRR3083908, SRR3083909, SRR3083910, SRR3083911, SRR3083912, SRR3083913, SRR2658589, SRR2658612, SRR2173784, SRR2173785, SRR2173786, SRR2173787, SRR5227280, SRR5227281, SRR6117986, SRR6117987, SRR6117988, SRR6117992, SRR6117993 and SRR6117994. The related publication is in the reference 5,13,14,15,16. Other datasets generated and analyzed for this study are available from the corresponding author on request.
