# Supplementary material for: Lateral confined growth of cells activates Lef1 dependent pathways to regulate cell-state transitions
Source: Sci Rep. 2022 Oct 15;12:17318. doi: 10.1038/s41598-022-21596-4 (PMC9569372; doi:10.1038/s41598-022-21596-4)
Supplement: Supplementary file 1 — Supplementary Information. [file 41598_2022_21596_MOESM1_ESM.docx]

**SUPPLEMENTARY FIGURES**

**Figure S1.**

(A) Differential interference contrast images of cells grown under lateral confinement for 4 and 6 days. (B) Confocal time-lapse image of the shape changes on Day 5 time point. (C) X-Z projection of the colonies in (B).

**Figure S2.**

Live and dead cells during the lateral confinement induced de-differentiation process. X-Z projection of colonies at Day2/4/10 time point. Hoechst 33342 (DNA) in cyan, DRAQ7 (dead cell) in magenta.

**Figure S3.**

(A) Confocal z-stack images of one day10 spheroid showing heterogeneity in Oct4 nuclear staining; scale bar is 20 um. (B) Boxplot showing increase of dead cell fraction in colonies during lateral confined growth. * P < 0.05, *** P < 0.001.

**Figure S4.**

(A) Heatmap showing log2 fold changes in gene expression of reprogramming factors. (B) This rearranged network from Fig. 2E shows the importance of Lef1 in the early de-differentiation stages. Proteins physically interacting with Lef1; the target genes of Lef1 were shifted up and separated from the remaining proteins by dashed lines. Transcriptional regulators that can be regulated by Lef1 are represented by blue borders.

**Figure S5.**

Boxplot showing increase of Oct4 positive nucleus in colonies during lateral confined growth. *** P < 0.001.

**Figure S6.**

(A) Quantitative qRT-PCR showing reduction in mRNA levels of Lef1 after siRNA knockdown. (B) Immunostaining and imaging of Lef1 shows reduction in protein levels on day5 under siRNA knockdown conditions. Scale bar is 20 um. (C) Boxplot showing significant reduction in Lef1 nuclear fluorescence intensity after siRNA knockdown as compared to control siRNA sample on day5; *** P < 0.001; n= 628, 722 nuclei for control and knockdown conditions, respectively. (D) Histogram and boxplot showing no difference in de-differentiation efficiency in control samples (without siRNA added) (n=1724) as compared to NC siRNA treatment (n=2109) measured by Oct4 nuclear fluorescence intensities. (E) Boxplot showing changes in de-differentiation efficiency (measured by Oct4 nuclear intensities) between Lef1 siRNA and control siRNA samples in another repeat experiment; n=2109, 2144, respectively. This siRNA targets exon 7 and 8. (F) Boxplot showing changes in Oct4 nuclear intensities between a different Lef1 siRNA and control siRNA samples. This siRNA targets exon 10 and 12.

**Figure S7.**

Alkaline phosphatase staining and qPCR shows reduced dedifferentiation efficiency in siRNA treated sample. (A) staining of the Day10 spheroid on the dishes. (B) higher resolution image showing that positive spheroids can be distinguished from negative stained ones. (C) Barplots shows the quantification of number of spheroids, area of the spheroids and the ratio of positive stained spheroids in control and Lef1 siRNA samples (data for three replicates are shown). Barplots shows standard deviation and the mean. (D) Barplots showing the fold change in expression level of Day4 Lef1 and Day10 Oct4 measured by qPCR.

**Figure S8.**

(A-C) Correlation coefficient on pixel wise basis for Lef1 and H3K9me3, Lef1 and H3K4me3, Lef1 and RNA pol II. ***P < 0.001.

**Figure S9.**

(A) List of investigated transcription factors, which could physically interact with Lef1. Table showing the number of targets and reprogramming factors shared between transcription factors and Lef1. (B, C) The effect of iCRT3 on de-differentiation efficiency measured by nuclear Oct4 fluorescence intensity (day10); n= 1971, 1589, 7251, 3693 respectively. (D) The effect of NFkB inhibitor SN50 on de-differentiation efficiency (day10); n= 1724, 2320 respectively.

**Figure S10.**

(A-C) the scatterplot shows the correlation among nucleus level of Lef1, cytoplasm level of GEF-H1 and α-Tubulin. Heatmap of gene expression changes of proteins in (D) actin cytoskeleton regulation pathway, (E) cell adherence junction pathway, and (F) WNT signaling pathway. Values are log2 fold change.

**SUPPLEMENTARY TABLE**

**Table S1**. Primers used for qRT-PCR and ChIP-qPCR

| Genes or sites | Forward | Reverse |
| --- | --- | --- |
| Lef1 | GTAGCTGAGTGCACGCTAAA | TAATTGTCTCGCGCTGACC |
| Oct4 | AGACCACCATCTGTCGCT | CAATGCTAGTTCGCTTTCTC |
| Nanog Promoter | CAAACCAAAAAGAGCCATTCAAGCTT | GTCGGCTTCTGTGTATAAGCAGA |
| Oct4 Promoter | CTTTGAGGAGAGGTGGAGAGCT | GCCTTGGCTGGACAATCCT |

**Table S2**. Sources of TF-target gene relationship.

* Harmonizome_ChEA_TFTarget-gene_attribute_edges.txt.gz (199 TFs)

* Harmonizome_ChEA_ChIP-gene_attribute_edges.txt.gz (333 ChIP studies)

* Harmonizome_ENCODE_TFTarget-gene_attribute_edges.txt.gz (181 TFs)

* Harmonizome_ENCODE_ChIP-gene_attribute_edges.txt.gz (1679 ChIP studies)

* Harmonizome_ESCAPE_Pubmed-gene_attribute_edges.txt.gz (84 ChIP studies)

* Harmonizome_TRANSFAC_Curated_TFTarget-gene_attribute_edges.txt.gz (201 TFs)

* Harmonizome_TRANSFAC_Predicted_TFTarget-gene_attribute_edges.txt.gz (158 TFs)

* Harmonizome_JASPAR_Predicted_TFTarget-gene_attribute_edges.txt.gz (111 TFs)

* Harmonizome_MotifMap_Predicted_TFTarget-gene_attribute_edges.txt.gz (329 TFs)

* Enrichr_ENCODE_TF_ChIP-seq_2015 (816 studies)

* Enrichr_ENCODE_and_ChEA_Consensus_TFs_from_ChIP-X (104 TFs)

* Enrichr_ChEA_2016 (645 redundant TFs)

* Enrichr_TRANSFAC_and_JASPAR_PWMs from Enrichr (326 redundant TFs)

**Table S3**. RNAseq datasets from NCBI-SRA databased used in this study.

| SRA ID | Description |
| --- | --- |
| SRR3083897 | RNASeq-E3.5ICM_rep1 |
| SRR3083898 | RNASeq-E3.5ICM_rep2 |
| SRR3083901 | RNASeq-E4.0ICM_rep1 |
| SRR3083902 | RNASeq-E4.0ICM_rep2 |
| SRR3083903 | RNASeq-E5.5Epi_rep1_part1 |
| SRR3083904 | RNASeq-E5.5Epi_rep1_part2 |
| SRR3083905 | RNASeq-E5.5Epi_rep2_part1 |
| SRR3083906 | RNASeq-E5.5Epi_rep2_part2 |
| SRR3083907 | RNASeq-E5.5VE_rep1 |
| SRR3083908 | RNASeq-E5.5VE_rep2 |
| SRR3083909 | RNASeq-E6.5Epi_rep1 |
| SRR3083910 | RNASeq-E6.5Epi_rep2 |
| SRR3083911 | RNASeq-E6.5VE_rep1_part1 |
| SRR3083912 | RNASeq-E6.5VE_rep1_part2 |
| SRR3083913 | RNASeq-E6.5VE_rep2 |
| SRR2658589 | RNASeq-E14_rep1 |
| SRR2658612 | RNASeq-E14_rep2 |
| SRR2173784 | RNASeq-E14_serum_rep1 |
| SRR2173785 | RNASeq-E14_serum_rep2 |
| SRR2173786 | RNASeq-E14_2i_rep1 |
| SRR2173787 | RNASeq-E14_2i_rep2 |
| SRR5227280 | RNASeq-F123_rep1 |
| SRR5227281 | RNASeq-F123_rep2 |
| SRR6117986 | RNASeq-MEF_Female_rep1 |
| SRR6117987 | RNASeq-MEF_Female_rep2 |
| SRR6117988 | RNASeq-MEF_Female_rep3 |
| SRR6117992 | RNASeq-MEF_Male_rep1 |
| SRR6117993 | RNASeq-MEF_Male_rep2 |
| SRR6117994 | RNASeq-MEF_Male_rep3 |

**Table S4**. Sequences of the Dicer substrate siRNA and target exons.

| Dicer substrate siRNA | Sequence | Target exon (for NM_010703) |
| --- | --- | --- |
| Lef1 siRNA 1 | GUGCGUCAAUGCUCAUUUUAACAACUG | 12 |
|  | GUUGUUAAAAUGAGCAUUGACGCAC |  |
| Lef1 siRNA 2 | CAUGAAAGCAUUCAGAGGCUUCUUAAU | 8 |
|  | UAAGAAGCCUCUGAAUGCUUUCATG |  |
| Lef1 siRNA 3 | GGCAUCAUUAUGUAGCCAGAGUAACUG | 3 |
|  | GUUACUCUGGCUACAUAAUGAUGCC |  |
